# Endoscopic Management of Foreign Bodies in the Upper Gastrointestinal Tract of Adults

**DOI:** 10.1155/2015/658602

**Published:** 2015-07-15

**Authors:** Chih-Chien Yao, I-Ting Wu, Lung-Sheng Lu, Sheng-Chieh Lin, Chih-Ming Liang, Yuan-Hung Kuo, Shih-Cheng Yang, Cheng-Kun Wu, Hsing-Ming Wang, Chung-Huang Kuo, Shue-Shian Chiou, Keng-Liang Wu, Yi-Chun Chiu, Seng-Kee Chuah, Wei-Chen Tai

**Affiliations:** Division of Hepato-Gastroenterology, Department of Internal Medicine, Kaohsiung Chang Gung Memorial Hospital and Chang Gung University College of Medicine, 123 Ta-Pei Road, Niao-Sung District, Kaohsiung 833, Taiwan

## Abstract

*Background*. Foreign object ingestion and food bolus impaction are a common clinical problem. We report our clinical experiences in endoscopic management for adults, foreign body ingestion, and food bolus impaction. *Method*. A retrospective chart review study was conducted on adult patients with foreign body ingestion and food bolus impaction between January 2011 and November 2014. Patients with incomplete medical records were excluded. * Results*. A total of 198 patients (226 incidents) were included in the study (male/female: 1.54/1; age 57 ± 16 years). Among them, 168 foreign bodies were found successfully (74.3%). 75.6% of the foreign bodies were located in the esophagus. Food bolus impaction was most common (41.6%). 93.5% of foreign bodies in current study cohort were successfully extracted and 5 patients required surgical interventions. Comparisons between symptomatic and asymptomatic patients revealed that locations of foreign bodies in the pharynx and esophagus were the significant relevant factors (*P* < 0.001). Shorter time taken to initiate endoscopic interventions increased detection rate (289.75 ± 465.94 versus 471.06 ± 659.93 minutes, *P* = 0.028). *Conclusion*. Endoscopic management is a safe and highly effective procedure in extracting foreign body ingestion and food bolus impaction. Prompt endoscopic interventions can increase the chance of successful foreign bodies' detection.

## 1. Introduction

Foreign bodies' ingestion and food bolus impaction are common clinical problems and are some of the most common endoscopic emergencies [1]. The majority of ingested foreign bodies pass spontaneously. Only 10~20% of cases require nonoperative intervention, and 1% or less require surgical procedures [2]. Fortunately, mortality as a result of foreign bodies' ingestion is extremely rare [2]. Foreign bodies' ingestion occurs more commonly in the pediatric population, taking up 80% of total ingestions, with a peak incidence from 6 months to 3 years [3–8]. For the 20% of ingestions that occur in adults, most take place while eating, leading to either bone or meat bolus impaction. [4]. On the other hand, true foreign body ingestion (i.e., nonfood objects) in adults occurs more commonly in individuals with psychiatric disorders, developmental delay, and alcohol intoxication and those seeking secondary gain [9]. Flexible endoscopy is the ideal choice for both diagnostic and therapeutic purposes in the management of upper GI tract foreign bodies with a success rate of over 95% and with minimal complications [4, 10]. Nevertheless, approximately 1500 Americans have died annually from foreign bodies in the upper gastrointestinal tract [11] and recent advances in endoscopic techniques could improve the survival rate. Foreign bodies' ingestion and food bolus impaction are also a common problem in Taiwan, but an endoscopic unit is not always readily available in many hospitals. In fact, there are limited reports on the application of flexible endoscopy in the management of ingested FBs and food bolus impaction for adults in Taiwanese literature. The aim of the current study was to report our clinical experiences in the endoscopic management of this study cohort.

## 2. Patients and Methods

### 2.1. Ethics Statement

This retrospective chart review study was approved by both the Institutional Review Board and Ethics Committee of Chang Gung Memorial Hospital, Taiwan (IRB104-0073B). The Ethics Committee waived the requirement for informed consent, and each patient's medical records were anonymized and deidentified prior to access. All patients provided their written informed consent before endoscopic interventions. None of our patients belonged to the minors/children age groups.

### 2.2. Patients

A retrospective chart review study was conducted on adult patients (age > 17 years) with suspected FB ingestion who visited the emergency department or outpatient clinic or during hospitalization in Kaohsiung Chang Gung Memorial Hospital, Taiwan, between January 2011 and November 2014. A total of 198 patients who met the criteria were included in the study. Patients with reported ingestions were first screened by an otolaryngologist before undergoing a digital esophagogastroduodenoscopy (EGD) examination with a flexible endoscope (GIF-XQ 40, GIFXQ 200, GIF-XQ 240, GIF-N230, etc.; Olympus Optical Co., Ltd., Tokyo, Japan) and were examined by radiology using either plain radiographies or computed tomography. We sorted the ingested foreign bodies into three groups according to their nature: food bolus, sharp FBs (i.e., fish and chicken bone), and blunt objects (i.e., coins and fruit stones). Depending on the nature and location of the foreign bodies, we used a wide diversity of endoscopic devices (retrieval basket, biopsy forceps, polypectomy snare, transparent distal protective hood, and overtube) to perform the procedures.

### 2.3. Methods

The clinical variables analyzed were age, sex, the type, number, and location of FB, associated upper gastrointestinal diseases, endoscopic methods, accessory devices, symptoms, and complications during the procedure. The mean time to endoscopy approach to foreign bodies is defined as the time interval when patients visited emergency department or outpatient clinics or during hospitalization and the time when the endoscopy procedure was done.

### 2.4. Statistical Analysis

Comparisons of continuous variables were conducted by Student's *t*-test. Categorical variables were compared using Pearson's *χ* ^2^ test. A 2-tailed significance value of 0.05 was used. All statistical analyses were performed using PASW statistical software, version 18 (IBM Co., Somers, NY).

## 3. Results

### 3.1. Patients' Demographic Characteristics

Of the 198 adult patients with suspected foreign bodies' ingestion, 78 were females (male/female: 1.54/1). The range of age at diagnosis was 18–90 years, with a median age of 57 ± 16 years.  Table 1 shows the age and gender distributions of foreign bodies' ingestion, which were similar across the three age classes.

### 3.2. Types and Location of Foreign Bodies

A total of 226 incidents of foreign bodies' ingestion occurred in the 198 patients in the current cohort. Of the 226 incidents, 168 foreign bodies were found, thus giving an FB detection rate of 74.3%. Only seven of the 58 patients who failed to detect foreign bodies returned to our clinic or emergency room for following up or due to persistent symptoms. All of them did not have major complications (such as perforation or bleeding) under surveillance, except one case who was incidentally diagnosed of esophageal cancer during endoscopic examination. The majority of foreign bodies found were food boluses, making up 41.6% of total FBs found by endoscope (Table 2). Fish bones were the second most frequent (12.5%) and dentures were third (7.7%). Other foreign bodies included coins, tablets with tinfoil packs, chicken bones, chopsticks, batteries, toothpicks, plastic rods, cotton balls, razor blades, glass, and crab shell. The esophagus was the most common lodgment site of ingested foreign bodies, taking up 75.6% of incidents (Table 3). Other lodgment sites were the stomach (12.5%), the pharynx (8.3%), anastomoses (2.4%), and the duodenum (1.2%), respectively. The majority of esophageal foreign bodies were found in the upper esophagus (59.1%) and midesophagus (26.8%). On the other hand, 58 incidents of reported foreign bodies' ingestion failed to be found by endoscopic procedures. The patients were reported to have ingested fish bones (*n* = 33, 56.9%), food boluses (*n* = 10, 17.2%), and dentures (*n* = 6, 10.3%).

### 3.3. Clinical Presentations after Foreign Bodies' Ingestion

The most frequent symptomatic complaint after foreign bodies' ingestion was odynophagia (36.5%). Other symptoms were dysphagia (27%), foreign body sensation (27%), chest pain (4.2%), nausea (3.1%), and others (2.2%). Thirty-four patients did not complain of any symptoms after foreign body ingestion and were classified as asymptomatic patients in contrast to symptomatic patients (*n* = 192). Among them, foreign bodies were most commonly located in the stomach and duodenum when found (17/34; 50%).

Foreign bodies were not found in 49 of the 192 symptomatic patients (25.5%), and fish bones (32/49) were the most commonly reported. Foreign bodies were most commonly lodged in the upper esophagus (*n* = 71, 37%) in the symptomatic patients (Table 4). The location of foreign bodies in the pharynx and esophagus was one of clinical factors relevant to the presentation of symptoms (*P* < 0.001).

### 3.4. The Detection Rate of Foreign Bodies by Plain Radiographies and Computed Tomography (CT)

The detection rate of foreign bodies by plain radiographies and CT was summarized in Table 5. One hundred thirty-one of 226 reported foreign bodies ingestion incidences were examined with only plain radiographies and 8 only with CT. Forty-eight of 226 incidences were examined by both plain radiographies and CT. Thirty-nine (17.2%) incidences were not examined by radiologist. The plain radiographies detection rate (sensitivity) for foreign bodies which was proven by endoscopy was 33.3% (44/131). The CT detection rate (sensitivity) for foreign bodies which was proven by endoscopy was 89.4% (34/38). The positive predictive value of plain radiographies was 77.1% (44/57). The positive predictive value of CT was 85% (34/40).

### 3.5. Timing of Endoscopic Interventions

Our results showed the average “time-to-scope” was 335.5 ± 526 minutes in current study. However, the mean time for successful localization of foreign bodies was shorter than that for unsuccessful localization (289.75 ± 465.94 minutes versus 471.06 ± 659.93 minutes, *P* = 0.028).

### 3.6. Endoscopic Management, Devices Used, and Complications

Out of the 168 incidents where foreign bodies were successfully identified, endoscopic removal was successful in 157 occasions (retrieval success rate 93.4%). The selection of endoscopic management methods and accessory devices depends on the location and type of foreign bodies ingested. The most common device used was biopsy forceps, for both food boluses and sharp foreign bodies (used in 51.2% of all identified foreign bodies), which achieved a 98.8% success rate of foreign bodies removal in any part of gastrointestinal tract. For sharp foreign bodies, we used a transparent distal hood to store the foreign bodies to avoid mucosal injury during endoscopic retrieval procedure. For esophageal food bolus impaction (61 incidents), the push technique (pushing the food bolus into the stomach) was applied on 15 occasions (24.6%), compared to the piecemeal extraction technique, which was applied on 45 occasions (73.8%). Both techniques were equally effective and no complications resulted in the current study.

The details of the 11 patients who failed the retrieval of foreign bodies were summarized in Table 6. One patient who ingested a razor blade was complicated by stomach perforation. He received surgical intervention immediately. Four patients who ingested fish bones underwent surgery because the fish bones were deeply embedded in the esophagus with high risk of perforations as shown by CT scans. The only procedure-related complication occurred to one patient during the retrieval process of a fish bone in the esophagus when the operator did not use any additional protective devise during the procedure. A deep mucosal injury was found and fortunately it was successfully closed by using hemoclips. The overall procedure-related complication rate for endoscopic retrieval of foreign bodies is 0.6 percent in the current study.

## 4. Discussion

In the current study, 168 out of 226 incidents of foreign bodies' ingestion (74.3%) were identified. The timing of the endoscopic procedure was the major factor influencing the detection rate of foreign bodies. In our series, fish bones, which are notorious for their elusiveness in both radiological and endoscopic studies, made up the majority of endoscopically undetectable FBs (33/58, 56.9%). Out of the 84 incidents of reported fish bone ingestion, only 51 of them (60.7%) were successfully localized endoscopically and fewer (*n* = 44, 52.3%) were detected by X-ray or CT. Four fish bone ingestion incidents needed surgical intervention due to penetration of the esophageal wall. Taiwan is an island with an abundant supply of fish, and it forms a major component of the daily diet of local inhabitants. Locals are accustomed to eating fish without removing the bones, and the latter are commonly present in broths or soups; this is likely to increase the chance of bone ingestion.

The timing of endoscopic procedure after the onset of foreign bodies' ingestion is an important factor influencing the outcome. In the current study, we observed that patients who had their foreign bodies successfully localized were those who took less time to initiate the endoscopic procedure, compared to those who failed (289.75 ± 465.94 versus 471.06 ± 659.93 min, *P* = 0.028). The average “time-to-scope” was 335.5 ± 526 min in the current study. Possible explanation for the delay could be that patients first visited local physicians before being referred to a tertiary center.

Most of the ingested foreign bodies were located in the esophagus (75.6%), especially in the upper esophagus. This finding is consistent with previous studies [12–15] and was probably because the ingested foreign objects commonly lodged in areas of the intestinal tract where the lumen was physiologically or pathologically narrow. The esophagus has four physically narrow areas which include the upper esophageal sphincter, the level of the aortic arch, the crossing of the main stem bronchus, and the lower esophageal sphincter [16]. Pathological narrowing of the intestinal tract due to underlying disease may also lead to food impaction [15, 17–19]. In our study, 66 out of 226 (29.2%) incidents of foreign bodies' ingestion were associated with underlying pathologies including esophageal and hypopharyngeal cancer, postcaustic injury benign stenosis, esophageal stricture, and anastomoses. As observed in the 127 esophageal food impaction incidents, 54 (42.5%) of them were associated with esophageal pathologies. Due to this association with possible esophageal disease, it is recommended that a routine repeat endoscopy be carried out after extraction of the foreign bodies to find out the possible occult underlying pathologies [12]. For those patients without underlying pathology (*n* = 73), sharp ingestions (55, 75.3%) such as fish bones, chicken bones, and dentures were the most common foreign bodies found. This was not surprising as these are sharp-pointed objects that could easily lodge in the lumen of a normal intestinal tract.

After foreign bodies' ingestion, the majority of patients (192/226 patients, 85%) were symptomatic, and only a few patients were asymptomatic. In our analysis, the majority of asymptomatic patients had their ingested foreign bodies located in the stomach or duodenum (50%), whereas most of the ingested foreign bodies were located in the upper esophagus (73/192, 38%) for the symptomatic patients. This is consistent with findings reported by Zhang et al. [13]. Almost all patients with fish and chicken bone ingestion were symptomatic (92/94). This was also not surprising because these are sharp-pointed foreign bodies that became easily lodged in the more sensitive upper esophagus. Foreign bodies' detection rate was not enhanced by the presence of symptoms (*P* = 0.907). This could be explained by the fact that most sharp-pointed foreign bodies may result in some degree of mucosal damage which becomes symptomatic during passage along the alimentary tract and possibly causes perforation, which ranged from 15% to 35% [20, 21]. Patients with underlying alimentary pathology were symptomatic regardless of the type of foreign bodies ingested. This may have contributed to the high foreign bodies' detection rate (62/66 incidents, 93.9%) among this group of patients. Computed tomography (CT) is much more sensitive in the detection of any kind of foreign bodies prior to endoscopic interventions than plain radiographies. Importantly, the role of CT scan is not only to localize esophageal foreign bodies but also to evaluate relevant regional complications such as perforation, fistulization, and even pleural empyema [22]. Zhu et al. reported that a more advanced dual source CT can even elevate the sensitivity and specificity to near 100% of diagnosis and evaluation of esophageal foreign bodies violently [23].

Different endoscopic methods and equipment are used according to the type and location of foreign bodies. In the current cohort, biopsy forceps (67.1%), retrieval basket (14%), and polypectomy snares (11.7%) were the most frequently used devices for FB retrieval. For sharp foreign bodies, an additional transparent distal hood to store the foreign bodies to avoid mucosal injury during endoscopic retrieval procedure is the key to the low procedure-related complication in the current study. All of them gave satisfactory results with near 100% success rate in any part of the gastrointestinal tract except for one patient with deep mucosal injury when the operator did not use any protection to the foreign body. Fortunately, the wound was successfully sealed off by hemoclips. Food boluses can usually be managed by en bloc removal using various grasping devices (e.g., polypectomy snare, retrieval net, friction-fit adaptor, or banding cap) or removal using a piecemeal approach [2]. Some experts advocate against the push technique, which involves pushing the bolus into the stomach without first examining the esophagus distal to the obstruction, under the notion that it may increase the risk of perforation due to the high incidence of esophageal pathology associated with food impactions [24]. In the current study, out of the 61 incidents of esophageal food bolus impaction, the push technique was applied on 15 occasions (24.6%) and the extraction technique on 45 occasions (73.8%). Both techniques were equally effective without any complications. These results are consistent with both series reported by Vicari et al. and Longstreth et al., respectively [25, 26].

Small objects (<6 cm, e.g., coins, battery, dentures, and plastic tubes) were managed by extraction, using the most suitable endoscopic accessories to grasp the objects. Biopsy forceps were used to grasp the edge of a coin that was located in the stomach in one case and a retrieval basket was used to extract an esophageal coin in another case; both were done successfully. As recommended by the American Society for Gastrointestinal Endoscopy guideline [2], a polypectomy snare was used for successful retrieval in many cases (plastic tube, denture, chopsticks, and toothpick) without complications.

## 5. Conclusion

Endoscopic management is a safe and highly effective procedure for extracting ingested foreign bodies and food bolus impaction. Prompt endoscopic interventions may increase the chance of successful foreign bodies' detection.

## Figures and Tables

**Table 1:** Demographic characteristics of patients with suspected foreign body ingestion.

Age (y)	Male (*n*)	Female (*n*)	Total
18–59	62	38	100
≥60	58	40	98

Total	120	78	198

**Table 2:** Types of foreign bodies ingested by a cohort of patients.

Types of foreign body	Detection by scope	Total	Detection rate
Food bolus	70	80	87.5%
Fish bones	51	84	60.7%
Chicken bones	9	10	90%
Coins	2	2	100%
Toothpicks	4	5	80%
Medicine tinfoil pack	3	4	75%
Dentures	13	19	68.4%
Battery	1	1	100%
Others	15	21	71.4%

Total	168	226	74.3%

**Table 3:** Anatomic location of foreign bodies in patients with suspected foreign body ingestion.

Location of foreign body	Incidents
Oropharynx	14
Esophagus	(127)
Upper esophagus	75
Middle esophagus	34
Lower esophagus	18
Stomach	21
Duodenum	2
Anastomosis	4

Total	168

**Table 4:** Comparisons between symptomatic and asymptomatic patients who ingested foreign bodies (incidents).

Characteristics	Symptomatic (*n*)	Asymptomatic (*n*)	*P *
Gender			
Male	124	18	0.195
Female	68	16	
Age			
<60 y old	96	22	0.114
≧60 y old	96	12	
Detection of foreign body			
Positive	143	25	0.907
Negative	49	9	
Location of foreign body (scope detected)			
Pharynx and esophagus	133	8	<0.001
Stomach and duodenum	10	17	
Types of FB			
Sharp FB	102	22	0.211
Others	90	12	

FB: foreign body.

**Table 5:** The detection rate of foreign bodies by plain radiographies and computed tomography (CT).

	Positive plain radiographies	Negative plain radiographies	Positive CT findings	Negative CT findings	Neither plain X-ray nor CT was done	Total
Scope (+) for foreign bodies	44	87	34	4	31	168

Scope (−) for foreign bodies	13	35	6	12	8	58

Overall	57	122	40	16	39	226

The plain radiographies detection rate (sensitivity) for foreign bodies: 33.3% (44/131).

The CT detection rate (sensitivity) for foreign bodies: 89.4% (34/38).

The positive predictive value of plain radiographies: 77.1% (44/57).

The positive predictive value of CT: 85% (34/40).

CT: computed tomography.

**Table 6:** Patients who failed the retrieval process of foreign body.

Patient numbers	Type of foreign body	Outcome	Management
1	Razor blade	Stomach perforation	Surgery
2	Sharp fish bone	Penetrating the esophageal wall with high risk of perforation	Surgery
3	Sharp fish bone	Penetrating the esophageal wall with high risk of perforation	Surgery
4	Sharp fish bone	Penetrating the esophageal wall with high risk of perforation	Surgery
5	Sharp fish bone	Penetrating the esophageal wall with high risk of perforation	Surgery
6	Food bolus	Passed into stomach	Observation
7	Small blunt fish bone	In stomach	Observation
8	Denture	In 3rd portion of duodenum	Observation
9	shell	In stomach	Observation
10	plum	In stomach	Observation
11	Sharp fish bone	Deep mucosal injury during retrieving procedure	Hemoclip
